# Dynamics of public health messaging and healthcare activity in children during the 2022 iGAS surge: an observational study in England

**DOI:** 10.1093/pubmed/fdaf163

**Published:** 2026-01-12

**Authors:** Alexandra L Creavin, Ruth Kipping, Alastair D Hay

**Affiliations:** Bristol Medical School, Canynge Hall, 39 Whatley Road, Bristol BS8 2PS, UK; Bristol Medical School, Canynge Hall, 39 Whatley Road, Bristol BS8 2PS, UK; Bristol Medical School, Canynge Hall, 39 Whatley Road, Bristol BS8 2PS, UK

**Keywords:** behaviour, children, communicable diseases

## Abstract

**Background:**

Public health messaging during infectious disease outbreaks can influence healthcare demand. The winter 2022 surge in Group A Streptococcus (GAS) in England provided an opportunity to examine the relationship between communications and National Health Service (NHS) activity, informing future strategies for resilience and risk communication.

**Methods:**

This observational study analysed UK Health Security Agency (UKHSA) invasive GAS (iGAS) notifications, NHS 111, General Practice (GP), and emergency department (ED) surveillance data, prescription records, internet searches, and media reports. Temporal associations were assessed descriptively, with weekly differences from winter averages calculated.

**Results:**

Following initial media reports and UKHSA messaging, internet search interest rose sharply (4%–63%). In the subsequent week, there were increases in NHS 111 contacts (fevers +256%, sore throats +953%), acute respiratory infection ED visits (+155%), GP pharyngitis consultations (+356%), and community penicillin prescriptions (+134%) compared to winter averages. Compared to the previous week, consultations for scarlet fever declined.

**Conclusions:**

This is the first study to link outbreak communications with system-wide NHS activity in real time. Messaging likely prompted appropriate care-seeking, but the rapid return to baseline and the low predictive value of consultations for iGAS suggest that many were for self-limiting illness. Findings highlight the need for tailored messaging, interdisciplinary collaboration, and scalable healthcare capacity during outbreaks.

## Introduction

Outbreaks of communicable diseases can place a significant strain on healthcare systems.^1^ Public health messaging is vital for controlling disease spread, offering guidance on prevention, transmission reduction, and accessing care, but its unintended consequences, such as overwhelming services, are less well understood.^2^

Group A Streptococcus (GAS) infections range from mild conditions like pharyngitis to severe invasive GAS (iGAS), which can be fatal.^3^ In the UK, iGAS is a notifiable disease, requiring clinicians to report suspected cases to the UK Health Security Agency (UKHSA) for timely surveillance and response (Supplementary Material A). iGAS is also notifiable in countries including Canada, Australia, and the USA.^4–9^

In autumn 2022, UKHSA reported rising notifications of scarlet fever and iGAS. Similar global trends were observed, possibly linked to postpandemic immune changes.^10–17^ By December, NHS primary care and emergency services faced overwhelming demand, alongside penicillin shortages.^18–21^ The Royal College of General Practitioners (RCGP) described winter 2022 as *‘one of the most challenging on record’*.^22^ These pressures followed public health messaging via UKHSA and Royal College press releases and blogs and national media reporting.

Media coverage and public health messages can rapidly alter healthcare-seeking behaviour; for example, extensive reporting of Jade Goody’s cervical cancer was followed by a surge in UK screening attendance.^23^^,^^24^

The aim of this study was to assess the temporal relationship between public health messaging and weekly changes in NHS activity related to GAS during the 2022 outbreak in England.

## Methods

### Design

This was an observational time-series comparison study.

### Population

This study included residents of England from NHS, RCGP, and UKHSA surveillance systems with confirmed iGAS infection, or potential GAS-related symptoms, including scarlet fever. The study population was primarily children aged 0–14 years, matching syndromic surveillance age groups. Data from RCGP surveillance, community prescribing, and NHS England monthly emergency department (ED) attendances covered all ages or were presented as rates per total registered population.

### Data

Data were obtained from UKHSA surveillance systems (HPZone, NHS 111, GP in-hours, ED syndromic data) and publicly available prescription data. RCGP data were extracted from publicly available reports. Most data were provided as aggregates International Organisation for Standardisation (ISO) week. The main period of analysis focused on the winter of 2022, specifically from November 2022 to January 2023, covering the iGAS surge peak and related messaging. Coverage periods varied by source.

### Data sources

#### Surveillance of Group A Streptococcus/invasive Group A Streptococcus cases

Aggregate weekly counts of confirmed iGAS cases in children aged 0–14 years were extracted from the UKHSA case management system (HPZone) from ISO week 1, 2017 to week 52, 2023. Confirmed cases are defined as GAS from a sterile site or severe nonsterile presentations.

#### Internet search and media data

Internet search patterns were analysed using Google Trends for UK-based searches of ‘scarlet fever’, ‘scarlet fever symptoms’, ‘Strep A symptoms’, ‘Strep A’, and ‘Strep throat’ within ‘All categories’, from 26 May 2019 to 26 May 2024. Daily standardized interest scores (0–100) were compared descriptively with the timing of media reports and official communications.

UKHSA press releases and blogs from 1 November 2022 to 15 May 2023 were sourced from the UKHSA website. First online news reports of paediatric deaths related to iGAS were identified using Google with keyword searches, and British Broadcasting Company (BBC) homepage searches for ‘Strep’ were conducted to gauge national media reporting. Reporting of Welsh child deaths was also noted due to the expected overlap.

#### Syndromic and healthcare data

Syndromic surveillance data from the UKHSA provided anonymized weekly counts for NHS 111 triaged calls, NHS 111 online usage, GP in-hours consultations, and ED visits.

#### NHS 111 data

Weekly triaged calls or online contacts coded as ‘fever’ or ‘sore throat’ in children aged 0–14 years (calls) and 5–14 years (online) were analysed from October 2020 onwards. Data for calls and online contacts were analysed together and separately.

#### Emergency department data

Weekly ED attendances for codes ‘scarlet fever’ and ‘acute respiratory infection’ (ARI) in children aged 0–14 years were obtained from 46 Type 01 ED units reporting to UKHSA from March 2018 to January 2024. These represent ~27.1% of Type 01 EDs in England, and data were converted per unit counts. ARI includes scarlet fever; both are shown separately. Monthly all-age ED attendance data for each winter (November–January) from 2018 to 2023 were extracted separately from NHS England reports.^25^

#### GP in-hours data

Weekly rates for codes ‘scarlet fever’ and ‘sore throat (pharyngitis)’ codes for children aged 0–14 were obtained from UKHSA GP in-hours syndromic surveillance from January 2017 to January 2024. This system monitors visits to GPs during regular surgery hours for specific syndromic indicators across England. Coverage increased over time from 5.76 million to 7.05 million.

Weekly incidence rates for codes ‘strep throat’ and ‘tonsillitis’ in all age groups were extracted from RCGP reports of provisional data for each winter from 2017/18 to 2022/23.^26^ Data were available as a rate per 100 000 population. Coverage gradually increased from 153 practices (2017) to 5013 (2023).

Monthly community prescription counts for penicillin V, amoxicillin, and clarithromycin for all ages were extracted from NHS England prescribing datasets for each winter from 2018/19 to 2023/24.^27^ Data are available as all-age monthly counts for England.

### Statistical methods

Analysis was prespecified and conducted in Stata 18 and Microsoft Excel. Descriptive analyses compared the timing of peaks in internet search interest with media communications, press releases, and blogs.

Weekly iGAS cases, NHS 111 contacts, and monthly prescription counts were expressed per 100 000 population using mid-year England estimates.^28^ Weekly ED attendances were converted to rates per reporting unit. Activity was plotted by ISO week, with winter means calculated for all available years (November–January, excluding 2020/21—Supplementary material B). An exploratory ‘predictive value’ (PV) was estimated as the ratio of confirmed iGAS notifications to GP in-hours consultations for pharyngitis or scarlet fever, indicating consultations associated with invasive disease.

## Results

### Media reports and public health communications

Between 24 November and 2 December 2022, three deaths in children under 10 attributed to GAS were reported in English and Welsh online news media (Supplementary material C). On 2 December (ISO week 48), following a fourth child death media report, UKHSA released a proactive media communication reporting five deaths in children under 10 and advising parents or guardians to ‘contact NHS 111 or your GP if you suspect your child has scarlet fever’.^29^ Symptoms and features indicating a need to contact health services were listed (Supplementary material D). This press release was quoted in the national media the same day.^30^ A further UKHSA blog on 5 December provided more details on the organism and actions for schools.^31^ Additional blog updates followed^29^^,^^31^ and a joint statement from the Royal Colleges was issued on 9 December.^32^  Supplementary material C details media reports and official communications chronologically.

### Internet searches

**Figure 1:**
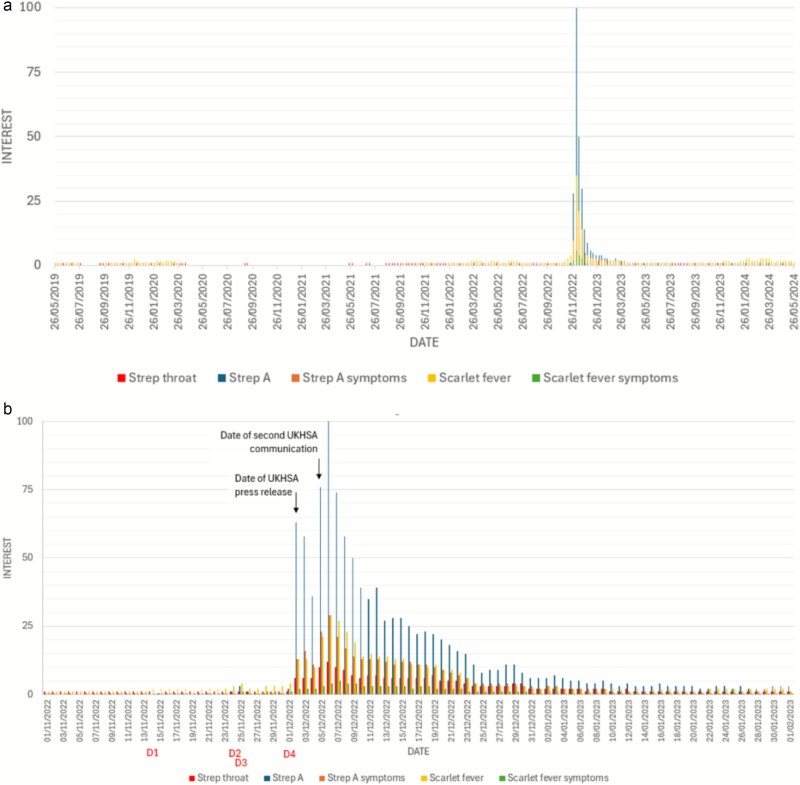
Internet searching behaviour. (a) Daily trend in Strep A–related Google searches in England from 26 May 2019 to 26 May 2024. (b) Magnified view of searches from 1 November 2022 to 1 February 2023.

Interest in all GAS-associated terms peaked in December 2022 compared with May 2019–24 (Fig. 1). Prior to the first UKHSA press release (2 December), media reports of deaths correlated with small rises in ‘Strep A’ searches up to 4% of the maximum. On December 2, a peak in searches for all strep-related terms occurred with the highest interest in the term Strep A (from 3% to 63% of maximum interest). In the following 2 days, interest remained high but fell to 31% of maximum interest. A second UKHSA communication on 5 December and subsequent media coverage (Supplementary material C) were associated with a rise in interest to 76% on the same day and 100% on the following day, alongside multiple BBC interviews and reports. After this, interest fell gradually despite a third UKHSA press release. The release of joint Royal College statements did not prompt an increase in interest. Despite an 11 January news report of further child deaths (Supplementary material C), interest did not rise above 5% of the maximum.

### Notifications of invasive Group A Streptococcus

**Figure 2:**
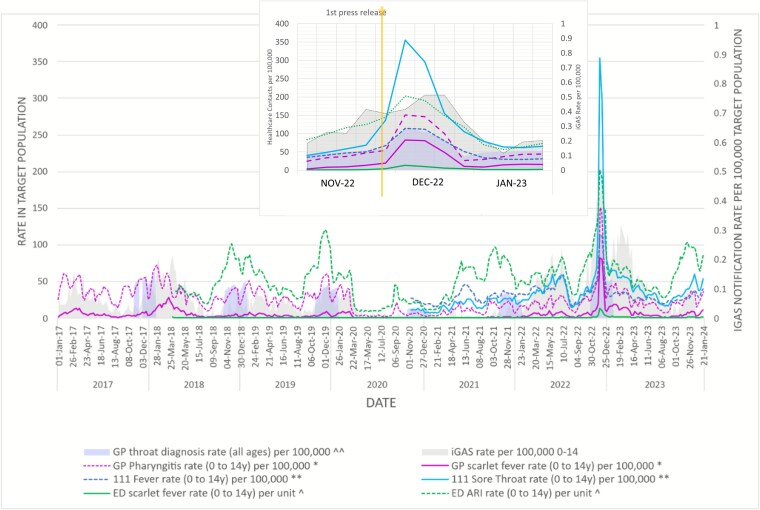
Weekly NHS healthcare activity related to potential symptoms for Strep A in 0- to 14-year-olds in England: January 2017 to December 2023.

Figure 2 presents weekly iGAS case rates from January 2017 to December 2023. During week 48 of 2022 (November 28), when public health messaging was issued, there were 0.4 iGAS cases per 100 000 in 0–14-year-olds in England, rising to 0.5 in weeks 50 and 51. This compares with a peak of 0.2 cases per 100 000 in previous years (2018 weeks 6 and 14). The 2022 annual rate in 0–14-year-olds was 7.6 cases per 100 000: compared to 3.0 in 2017, 3.7 in 2018, and 6.1 in 2023.

### Healthcare use

Figure 2 presents healthcare use rates for data available from January 2017 to December 2023. Table 1 summarizes the mean and peak rates. All health services saw increases in week 49 of 2022 (5–11 December), ranging between 139% and 2640% compared to the mean in winter means.

**Table 1:** Mean winter rate and peak rate of potential GAS-related contacts to NHS services.^a^

Surveillance system (weekly measure)	Indicator(s)	Age	Peak ISO^b^ week 2022	Peak value, 2022	Winter mean (95% CI)	Difference between week 49, 2022 & winter mean (%)
**RCGP surveillance** **(rate per 100 000 registered population^^**)	Strep throat & peritonsillar	All ages	49	22.1	1.2 (1.0,1.5)	+20.9 (+1742%)
	Tonsillitis & acute pharyngitis	All ages	49	92.4	38.6 (36.0,41.3)	+53.7 (+139%)
UKHSA GP in-hours syndromic surveillance (consultations per 100 000 registered population^*^)	Pharyngitis	0–14	49	151	33.1 (29.6, 36.6)	+118.1 (+356%)
	Scarlet fever	0–14	49	82.4	4.3 (3.7, 5.0)	+78.1 (+1816%)
**UKHSA remote health advice syndromic: NHS 111 (contacts** per 100 000 population ^**^)	Fever	0–14	49	115	32.1 (30.7, 33.6)	+82.4 (+258%)
	Sore throat	0–14	49	356	33.8 (29.3, 38.2)	+321.7 (+953%)
**UKHSA ED syndromic surveillance (attendances per ED^)**	Scarlet fever	0–14	49	13.7	0.5 (0.4, 0.6)	+13.2 (+2640%)
	Acute respiratory infection	0–14	49	203	79.6 (74.3, 84.9)	+123.2 (+155%)

a ^^Source: RCGP surveillance datahttps://www.rcgp.org.uk/representing-you/research-at-rcgp/research-surveillance-centre/public-health-data. (Royal College of General Practice, 2024) ‘Strep throat & peritonsillar’ and ‘Tonsillitis & acute pharyngitis’ diagnoses in all age groups across England combined. Data were available as a rate per 100 000 population. Coverage gradually increased from 153 practices in 2017 to 5013 practices in 2023. These data were not available by age. ^*^ Source: UKHSA surveillance data. All diagnoses coded as ‘scarlet fever’ and ‘sore throat (pharyngitis)’ in children aged 0–14 years supplied by in-hours General Practices across England. Supplied as a weekly rate per 100 000 registered population. Population coverage increased over time from 5.76 million in 2017, week 1, to 7.05 million in 2024, week 10, through recruitment of new GP practices and expansion of practices already participating. ^**^Source: UKHSA surveillance data. All NHS 111 calls or online contacts coded as primarily ‘fever’ and as primarily ‘sore throat’ in children aged 0 to 14 years by 111 practitioners. Only one code is applied to each consultation. Supplied as a weekly count and transformed into a rate using Office of National Statistics (ONS) mid-year target populations. ^Source: UKHSA surveillance data. All diagnoses coded as ‘scarlet fever’ and ‘acute respiratory infection’ in children aged 0–14 years by EDs across England. Reported by the same 46 Type 01 (Major) units, representing 27.1% of Type 01 EDs (out of *n* = 170). Supplied as a count and transformed into a rate per unit. ARI incorporates scarlet fever, but both are shown to illustrate the proportion of ARI diagnosed as scarlet fever.

b ISO week, numbered 1–52/53 each year.

#### UK Health Security Agency GP in-hours consultations

In ISO week 49, 2022, the in-hours GP consultation rate for scarlet fever in 0–14-year-olds was 82.4 per 100 000, 1816% higher than the winter mean [4.3 (95% CI 3.7, 5.0)]. The pharyngitis consultation rate peaked the same week at 151 per 100 000, 356% higher than the winter mean [33.1 (29.6, 36.6)] and 111% higher than the peak weekly rate in the 2017/18 peak season (71.7).

#### Royal College of General Practitioners GP consultations

Recording of throat infections in primary care (all ages) peaked in December 2022. Peak presentations for strep throat and peritonsillar illness (all ages) occurred in ISO week 49, 2022, at 22.1 per 100 000 (Supplementary material E), 1742% higher than the mean weekly winter presentation rate. Tonsillitis and acute pharyngitis peaked in week 49 at 92.4 per 100 000, 139% higher than the mean weekly winter rate.

#### Community pharmacy prescriptions

**Figure 3:**
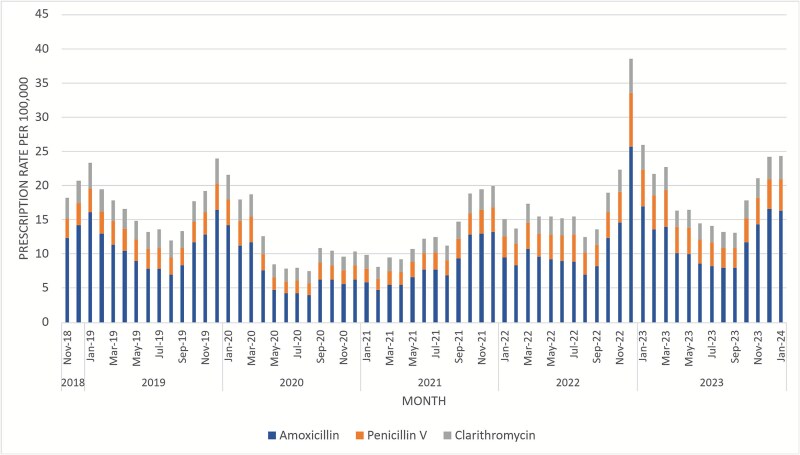
Monthly all-age community dispensing rate of penicillin V, amoxicillin, and clarithromycin per 100 000 population: England, November 2018 to January 2024.

In December 2022, penicillin V community prescriptions for all ages were 134% higher than the winter mean (Fig. 3). Penicillin V shortages led to shifts to amoxicillin (90% rise) and clarithromycin (56% rise) prescriptions compared with winter means in December 2022.

#### NHS 111 contacts (triaged calls and online assessments)

Total NHS 111 weekly contact rates for fever in 0–14-year-olds peaked at 115 per 100 000 in week 49, 2022, a 258% increase from the winter mean [32.1 (95% CI 30.7, 33.6)] (Table 1). Sore throat contact rates reached 356 per 100 000, 953% above the winter average [33.8 (29.3, 38.2)]. The largest proportionate increase was in online assessments.

#### Emergency department attendances

Weekly UKHSA surveillance data showed ED attendances for ARIs in children aged 0–14 peaked at 203 per unit in week 49, 2022, a 69% increase from the 2019 peak and 155% above the winter mean [79.6 per unit (95% CI 74.3, 84.9)]. Scarlet fever attendances increased by 2640% to 13.7 per 100 000 [winter mean 0.5 (95% CI 0.4, 0.6)].

Monthly NHS England data showed that Type 3 (minor injuries) EDs had the largest proportionate increase in total attendances (+20% across all ages) in December 2022; Type 1 EDs saw a +10% increase (Supplementary Material F).

#### Predictive value: ratio of invasive Group A Streptococcus activity to cases

An exploratory analysis of the ratio of iGAS cases to GP pharyngitis consultations showed high variability across years, reflecting the rarity of iGAS relative to the large number of sore throat consultations: while the rate of GP pharyngitis appointments rose in week 49, 2022 (PV = 1.8 × 10^−3^), higher ratios were seen in previous years since 2017, primarily due to the low weekly numbers of iGAS cases. Increases in consultations did not consistently correspond to higher iGAS case detection.

### Healthcare use and interest

Google search interest peaked on Friday, 2 December, coinciding with the UKHSA announcement and its media coverage. As this fell on a Friday, any effect on GP consultations would not appear until the following week (commencing 4 December), whereas NHS 111 activity could have been influenced over the weekend, though the week’s averages combine pre- and postannouncement data.

In the week of 4 December, GP consultations for pharyngitis tripled (54.2–151.2 per 100 000) before falling to 146.2 and then 100.4 in subsequent weeks. In contrast, GP scarlet fever consultations declined steadily from 82.4 per 100 000 (week of 27 November) to 80.5, 48.7, and 10.8 over the following 2 weeks despite further media coverage (Supplementary material C).

NHS 111 contacts for fever and sore throat almost doubled between the week of 27 November (67.1 and 68.5 per 100 000) and the week of 4 December (114.5 and 136.9).

## Discussion

### Main findings of this study

In early December 2022, England experienced a surge in scarlet fever and iGAS cases exceeding prior peaks since 2017. Following initial public health announcements and national media coverage, public interest in Streptococcus A terms surged, coinciding with substantial increases in NHS activity. NHS 111 contacts for fevers and sore throat rose sharply, especially online, despite the service not covering children under 5. ED visits for respiratory infections, GP consultations for pharyngitis, and penicillin V prescriptions also increased. Temporal patterns showed immediate rises in attendances for pharyngitis, fever, and sore throat the week following the announcements but not scarlet fever, suggesting that public health messaging and media coverage influenced health-seeking behaviour during the outbreak.

### What is already known on this topic?

The iGAS surge aligns with reports from England in 2022^17^^,^^33^ and accounts of overwhelmed general practices.^18^^,^^21^^,^^34^ The role of timely, reliable public health communication is well established, particularly given social media’s capacity to spread both accurate and misleading information.^35–39^ Media coverage is known to shape public behaviour,^40^^,^^41^ with documented effects ranging from increased cervical cancer screening uptake after celebrity diagnoses to rising suicide rates linked to reporting.^24^^,^^42^^,^^43^ In this outbreak, growing media interest may have influenced the timing of official communications.

### What this study adds

This study quantifies the timing and magnitude of NHS activity changes following national iGAS communications, showing rapid, system-wide increases across NHS 111 (+953% sore throat), GP consultations (+356% pharyngitis), and prescriptions (+134%) within a week of initial announcements. These findings directly link public health messaging to measurable shifts in service use.

The findings highlight both the protective role of timely communications in prompting appropriate care and the risk of disproportionate service use for self-limiting illness. Clear, equitable messaging aligned with service capacity is essential to safeguard public health and NHS sustainability.

The temporal link between announcements, media coverage, and NHS activity supports the view that such messaging effectively stimulates health-seeking behaviour. Much of the rise in consultations likely reflected appropriate responses to increased GAS/iGAS activity, with messaging potentially aiding early identification and reducing transmission. However, despite continued iGAS circulation later in December 2022, attendance rates quickly returned towards usual levels. This pattern may reflect the lag between symptomatic infection and iGAS notification and increasing public understanding of illness severity following early messaging. The lack of correlation between consultation rates and confirmed iGAS cases in the exploratory predictive value analysis suggests that rising consultations were not solely due to natural disease progression. The initial surge likely included many milder, self-limiting cases, contributing to temporary system strain. The marked rise in NHS 111 online use suggests potential saturation of call services.

We are not aware of prior studies examining this dimension of outbreak communication, making these findings valuable for informing closer collaboration between risk communicators, health providers, and the media.^44^ Alternative explanations for the observed increase include cocirculating respiratory infections, such as influenza, with overlapping symptoms.^45^

Messaging and media may also have lowered thresholds for antibiotic prescribing and shaped diagnostic labelling, increasing referrals, and amplifying system burden. Further work is needed to evaluate how public health messaging affects professional decision-making and risk appetite.

Predictable surges in public concern and media coverage during outbreaks may exaggerate apparent incidence through increased healthcare activity, creating noise in surveillance systems, and cautious interpretation of time series data is needed.

#### Future research

##### Message content and targeting

Research should assess how official, coproduced, tailored, and literacy-sensitive messaging—using visual guides—affects trust, care-seeking, and risk perception.^46^^,^^47^ Spatial analysis of search activity could be used to explore local responsiveness to outbreak messaging. Future outbreak evaluations could integrate data from public information and communication sources to assess message reach and impact.

##### Message adaptability

Studies should explore how messaging can flexibly respond to NHS pressures through improved dialogue including with frontline providers and targeted delivery.

##### System preparation

Investigate early warning systems, scalable NHS capacity, and how search trends and qualitative insights support outbreak preparedness.

### Limitations of this study

A key strength is the comprehensive analysis of high-coverage, multi-faceted healthcare utilization data and media reporting from across England over several years, offering high generalizability to outbreaks in comparable settings.

However, limitations arise because the data were not collected specifically for this research question. Syndromic surveillance indicators for ARI, fever, and sore throat may include consultations for other respiratory pathogens prevalent during winter 2022/23, such as influenza. Changes in health care access, usage patterns (e.g. increased awareness and use of NHS 111 online), and surveillance coverage over time (like the expansion of RCGP data collection) could influence trends. Unavailable out-of-hours primary care data for winter 2022 due to a technical issue may underestimate service impact. Delays in iGAS case reporting are possible, though typically short. While using confirmed iGAS cases as an outcome reduced bias, ascertainment bias remains a potential factor.

Google Analytics captures relative, sampled search volumes from Google (89%–94% of UK searches^48^) and may vary between extractions. UK-wide data proxy English interest but may include activity from other nations. Results depend on specific terms, weekly aggregation, and English language, potentially under-representing groups or obscuring exact timing.

## Conclusion

This is the first study to link outbreak communications with system-wide NHS activity in real time. Messaging likely prompted appropriate care-seeking, but the rapid return to baseline and low predictive value of consultations for iGAS suggest that many were for self-limiting illness. Findings highlight the need for trusted, tailored messaging, interdisciplinary collaboration, and scalable healthcare capacity during outbreaks. to reduce unnecessary consultations while ensuring timely care for those most at risk.

## Supplementary Material

Supplementary_material_A_fdaf163

Supplementary_material_B_fdaf163

Supplementary_material_C_fdaf163

Supplementary_material_D_fdaf163

Supplementary_material_E_fdaf163

Supplementary_material_F_fdaf163

## Data sharing

Where data isn't obtained from freely accessible sources, data sharing is restricted by institutional policy of the institution supplying the data.

## Notes

**Alexandra L. Creavin**, Clinical lecturer in public heath

**Ruth Kipping**, Associate professor of public health

**Alastair D. Hay**, Professor of primary care
