# Supplementary material for: Dynamics of public health messaging and healthcare activity in children during the 2022 iGAS surge: an observational study in England
Source: J Public Health (Oxf). 2026 Jan 12;48(1):281–90. doi: 10.1093/pubmed/fdaf163 (PMC13017340; doi:10.1093/pubmed/fdaf163)
Supplement: Supplementary_material_A_fdaf163 [file supplementary_material_a_fdaf163.docx]

# Supplementary material A: Typical NHS care pathways leading to notification of confirmed**^[[1]](#footnote-1)^** invasive Group A Streptococcus (iGAS) infection at the relevant regional Health Security Agency (UKHSA) **^[[2]](#footnote-2)^** **^[[3]](#footnote-3)^**


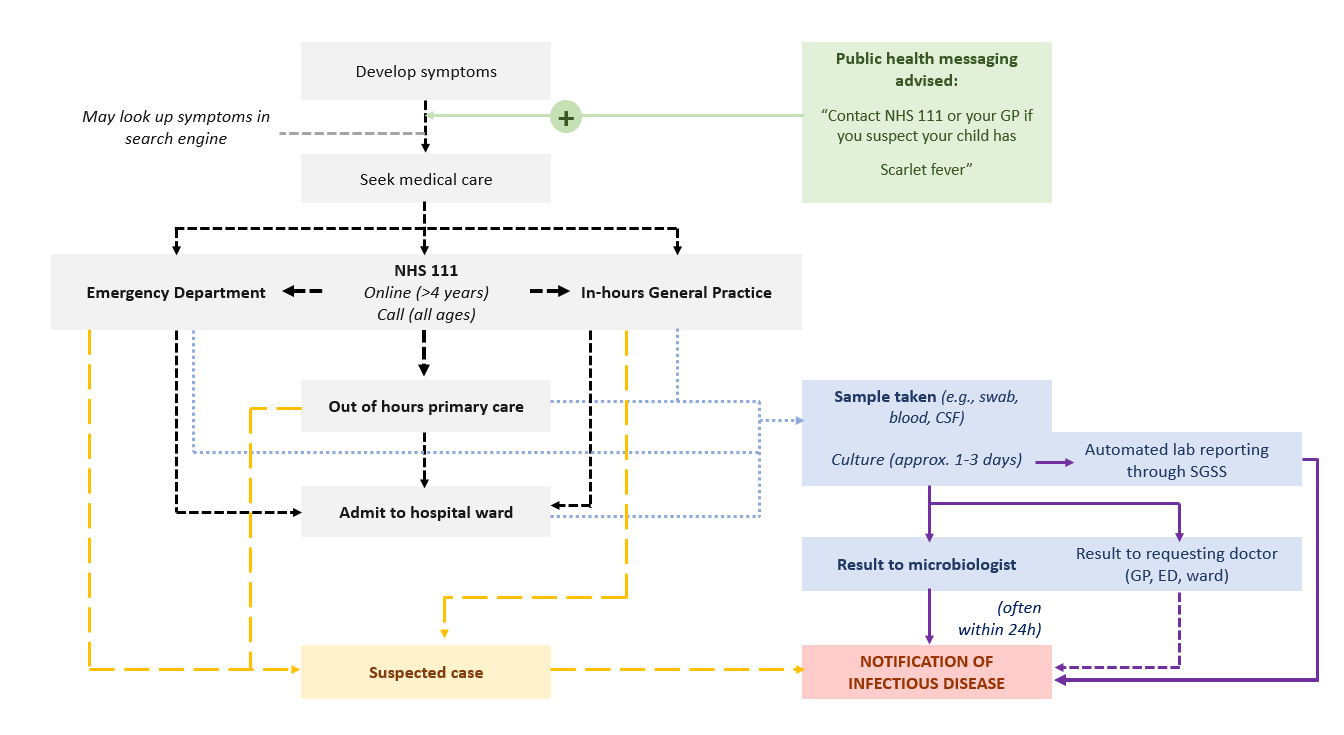
Alt text: Diagram illustrating the typical pathway from symptom development to notification of iGAS in England.

1. A confirmed case is a patient found to have Group A Streptococci in a normally sterile site. It also includes severe GAS infections, where GAS has been isolated from a normally non-sterile site (e.g., throat), in combination with a severe clinical presentation, (e.g., pneumonia). [↑](#footnote-ref-1)
2. ED = Emergency department, GP = General Practice. [↑](#footnote-ref-2)
3. [Notifiable diseases and causative organisms: how to report - GOV.UK (www.gov.uk)](https://www.gov.uk/guidance/notifiable-diseases-and-causative-organisms-how-to-report) [↑](#footnote-ref-3)
