# Supplementary material for: Dynamics of public health messaging and healthcare activity in children during the 2022 iGAS surge: an observational study in England
Source: J Public Health (Oxf). 2026 Jan 12;48(1):281–90. doi: 10.1093/pubmed/fdaf163 (PMC13017340; doi:10.1093/pubmed/fdaf163)
Supplement: Supplementary_material_B_fdaf163 [file supplementary_material_b_fdaf163.docx]

# Supplementary material B: Range of years used to calculate each winter mean

| **Winter mean** | **Years excluded** | **Years included** |
| --- | --- | --- |
| NHS 111 calls & online | 2020/21 | 2021/22 & 23/24 |
| GP mean (RCGP data) | 2020/21 | 2017/18 to 21/22 |
| GP mean (UKHSA data) | 2020/21 | 2017/18 to 21/22 |
| ED weekly | 2020/21 | 2018/19 to 21/22 & 2023/24 |
| ED monthly (by department) | 2020/21 | 2018/19 to 21/22 |
| Monthly prescriptions | 2020/21 | 2018/19 to 2021/22 |
