# Supplementary material for: Dynamics of public health messaging and healthcare activity in children during the 2022 iGAS surge: an observational study in England
Source: J Public Health (Oxf). 2026 Jan 12;48(1):281–90. doi: 10.1093/pubmed/fdaf163 (PMC13017340; doi:10.1093/pubmed/fdaf163)
Supplement: Supplementary_material_C_fdaf163 [file supplementary_material_c_fdaf163.docx]

# Supplementary material C: Reports of UK iGAS deaths by online news media and press releases from UKHSA and Royal Colleges relating to the rise in iGAS cases and child deaths from November 2022 to January 2023

| Date | ISO^[[1]](#footnote-1)^ week | Media source | Cumulative deaths reported | Location of deaths |
| --- | --- | --- | --- | --- |
| 24.11.22 | 47 | Online local news: The Metro (Robertson, 2022)  (Also reported by local BBC news on 25.11.22) | 1 | Surrey, England |
| 25.11.22 | 47 | Online local news: The Bucks Free Press (Perrin, 2022)  National news: The Telegraph (Telegraph Reporters, 2022), The Guardian (PA Media, 2022), and Sky News (SkyNews, 2022).  With a local BBC report on 5.12.22 (BBC Beds, 2022) | 2  (Death 14.11.22) | High Wycombe, Buckinghamshire, England |
| 25.11.22 | 47 | Online local news: MyLondon.News (Byrne, 2022)  With a local BBC report on 2.12.22 | 3 | Ealing, London, England |
| 1.12.22 | 48 | Online local news: BBC News Wales (Anonymous, 2022) with further local reports on 2.12.22 (BBC Wales, 2022b), 5.12.22 mentioning 8 deaths (BBC Wales, 2022d, 2022a), and 14.12.22 (BBC Wales, 2022c) | 4 | Penarth, Wales |
| 2.12.22 | 48 | UKHSA press release 1 (UKHSA, 2022b)  Covered by National News media: The Guardian, The BBC (BBC Health, 2022a), and in local media: The Hampshire Chronicle.(Murray & Davis, 2022)  UKHSA appearance on BBC news.(BBC Health, 2022c) | 5 | One in Hampshire, England |
| 3.12.22 | 48 | BBC summary of Newspaper front page headlines states that “Several” papers including the Daily Mail and have led with “the recent deaths of six young children who had contracted the Strep A bacterial infection in England and Wales.” (BBC News, 2022)  Two BBC articles about a sick child were published advising parents to “Get your child checked” with an update on the same child on 9.12.22 and 14.12.22.(BBC Health, 2022d, 2022e; BBC Manchester, 2022b; BBC UK, 2022) | NFR^[[2]](#footnote-2)^ | n/a |
| 5.12.22 | 49 | UKHSA press release 2 (UKHSA, 2022a)  BBC reports on the Hampshire death as 8^th^ death (BBC Hampshire & Isle of Wight, 2022a; BBC Health, 2022f) with a further article on 14.12.22 (BBC Hampshire & Isle of Wight, 2022b)  House of Lords question on Strep A reported in BBC (BBC Politics, 2022) | BBC article mentions 8^th^ child death but describes Hampshire death already reported. | n/a |
| 6.12.22 | 49 | Local BBC interview with Cambridge virologist (BBC Cambridgeshire, 2022)  Local BBC interview with Welsh parent (BBC Wales, 2022f)  Local BBC reports surge in ED attendance in Essex (BBC Essex, 2022a) | Article mentions 9 UK child deaths without details | n/a |
| 7.12.22 | 49 | Local BBC reports on ill child in Dorset (BBC Dorset, 2022)  Local BBC reports Strep A antibiotic shortage in Wales (BBC Wales, 2022h) and school scarlet fever outbreak (BBC Wales, 2022g)  National BBC reports good supply of Strep A antibiotics (BBC Health, 2022f) | NFR | n/a |
| 8.12.22 | 49 | UKHSA press release 3 (UKHSA, 2022b)  Local BBC reports that consultant advises ED is overcrowded in Essex (BBC Essex, 2022)  Local BBC reports Strep A antibiotic shortage in Manchester (BBC Manchester, 2022a) | NFR | n/a |
| 8.12.22 | 49 | RCGP request for clear advice for parents of children with suspected Strep A (RCGP, 2022b) and this is reported by national BBC (BBC Health, 2022g) | NFR | n/a |
| 9.12.22 | 49 | RCGP/RCPCH/RCEM joint statement (RCGP, 2022a)  National BBC article on Strep A symptoms (BBC Health, 2022h)  Local BBC: Secondary school death reported in Brighton (BBC Sussex, 2022) | Article mentions 16 UK child deaths with detail of 1 | East Sussex, England |
| 9.12.22 | 49 | RCGP Guidance announcement (RCGP, 2022b) | NFR | n/a |
| 12.12.22 | 50 | Local BBC: Strep A testing in Wales (BBC Wales, 2022f) | NFR | n/a |
| 12.12.22 | 50 | Local BBC: Strep A outbreak in Devon school (BBC Devon, 2022) | NFR | n/a |
| 13.12.22 | 50 | Local BBC: reports antibiotic shortage in Shropshire (BBC Shropshire, 2022) and 18,000 111 call in one weekend in Wales (BBC Wales, 2022e) | NFR | n/a |
| 14.12.22 | 50 | National BBC: Strep A advice (BBC Health, 2022i) | NFR | n/a |
| 15.12.22 | 50 | UKHSA updates (UKHSA, 2022b)  *Weekly till 19/1/23, fortnightly till 3/3/23, monthly till 15/5/23*  BBC Scotland: antibiotic shortages (BBC Scotland, 2022)  National BBC: alternative antibiotics (BBC Health, n.d.) | NFR | n/a |
| 16.12.22 | 50 | National BBC: reducing Strep A risk (BBC Health, 2022h) | Mentions 18 UK deaths |  |
| 28.12.22 | 52 | Local BBC: arrest of woman after death of child (BBC Wales, 2022) | Mentions 1 death from 22.12.23 | Lampeter, Wales |
| 30.12.22 | 52 | National BBC: Overview of strep deaths since mid-September (BBC Health, 2022a) | Mentions 30 UK deaths since mid-September | n/a |
| 11.1.23 | 2 | Local BBC: two child deaths over Christmas | 2 | Coventry, England |

Table references:

Anonymous. (2022, December 1). Strep A: Penarth primary school child dies of disease. *BBC News Wales*. https://www.bbc.co.uk/news/uk-wales-63828857#:~:text=A primary school child has,action to take if needed

BBC Beds, H. & B. (2022). Strep A: Fundraiser in Muhammad Ibrahim Ali memory reaches £4,500. *BBC*.

BBC Cambridgeshire. (2022, December 6). Strep A: What symptoms should we look for? *BBC*.

BBC Devon. (2022, December 13). Strep A: Major outbreak closes Kingsbridge school. *BBC*.

BBC Dorset. (2022, December 7). Dorset boy in coma after strep A misdiagnosis, family says. *BBC*. https://www.bbc.co.uk/news/uk-england-dorset-63857673

BBC Essex. (2022a, December 6). Strep A: Surge in parents at Broomfield A&E over child health concerns. *BBC*.

BBC Essex. (2022b, December 8). Strep A: Broomfield Hospital doctor gives advice to worried parents. *BBC*. https://www.bbc.co.uk/news/av/uk-england-essex-63904284

BBC Hampshire & Isle of Wight. (2022a). Waterlooville: Call for awareness after eighth strep A child death. *BBC*.

BBC Hampshire & Isle of Wight. (2022b, December 14). Strep A: Parents say son misdiagnosed before death. *BBC*.

BBC Health. (n.d.). Strep A: Pharmacies can now give alternative antibiotics. *2022*.

BBC Health. (2022a). Six children die with Strep A bacterial infection. *BBC*.

BBC Health. (2022b). Strep A: At least 30 children have died in UK since mid-September. *BBC*.

BBC Health. (2022c, December 2). Strep A: How common is it and what are the symptoms to look for? *BBC*. https://www.bbc.co.uk/news/av/health-63839585

BBC Health. (2022d, December 3). Father of girl, 4, fighting for life with Strep A infection is “praying for a miracle.” *BBC*.

BBC Health. (2022e, December 3). Strep A: “Get your child checked out” says father of four-year-old patient. *BBC*. https://www.bbc.co.uk/news/av/health-63847866

BBC Health. (2022f, December 5). Strep A death of eighth child confirmed. *BBC*. https://www.bbc.co.uk/news/health-63860028

BBC Health. (2022g, December 7). Antibiotics for strep A in good supply, says health secretary Steve Barclay. *BBC*.

BBC Health. (2022h, December 8). GPs say parents need clearer strep A advice. *BBC*. https://www.bbc.co.uk/news/health-63903051

BBC Health. (2022i, December 9). What is strep A and what are the symptoms? *BBC*. https://www.bbc.co.uk/news/health-63836093

BBC Health. (2022j, December 14). Strep A symptoms: What you need to know, in a minute. *BBC*.

BBC Health. (2022k, December 16). Flu nasal spray vaccine for children may reduce strep A risk. *BBC*.

BBC Manchester. (2022a). Strep A: Cheshire pharmacies report amoxicillin and penicillin shortages. *BBC*.

BBC Manchester. (2022b, December 14). Strep A: Bolton girl, 4, out of intensive care, mum says. *BBC*.

BBC News. (2022, December 3). Newspaper headlines: Strep A warnings and Matt Hancock diaries. *BBC*.

BBC Politics. (2022, December 5). Lords question on scarlet fever and strep A infections. *BBC*.

BBC Scotland. (2022, December 15). Strep A antibiotics supply alert issued. *BBC*.

BBC Shropshire. (2022, December 13). Pharmacists run out of penicillin as strep A demand soars. *BBC*.

BBC Sussex. (2022, December 9). Hove secondary school pupil dies with suspected strep A. *BBC*. https://www.bbc.co.uk/news/uk-england-sussex-63922734

BBC UK. (2022, December 9). Strep A: Grandmother says four-year-old Camila Burns getting better. *BBC*.

BBC Wales. (2022a). Strep A: Doctor urges parents to watch out for symptoms. *BBC*. https://www.bbc.co.uk/news/av/uk-wales-63864079

BBC Wales. (2022b). Strep A: Family of Penarth girl Hanna Roap “broken” by death. *BBC*.

BBC Wales. (2022c). Strep A links investigated after child’s death in Powys. *BBC*.

BBC Wales. (2022d). Strep A: Penarth family traumatised by daughter’s death. *BBC*.

BBC Wales. (2022e). Strep A: Woman arrested after girl, 8, dies in Lampeter. *BBC*.

BBC Wales. (2022f, December 6). Strep A: Mum warns of symptoms after child’s illness. *BBC*.

BBC Wales. (2022g, December 7). Strep A among Carmarthenshire school scarlet fever o. *BBC*.

BBC Wales. (2022h, December 7). Strep A: Antibiotics shortage in some Welsh pharmacies. *BBC*.

BBC Wales. (2022i, December 12). Strep A Q&A: Are tests available on the NHS in Wales? *BBC*.

BBC Wales. (2022j, December 13). Strep A: Weekend sees 18,000 calls to NHS Wales 111 service. *BBC*.

Byrne, F. (2022, November). Ealing child dies from invasive strep A with pupil at nearby primary school also in hospital after scarlet fever “outbreak.” *MyLondon.News*.

Murray, J., & Davis, N. (2022, December 4). Strep A: fears NHS will struggle to cope as seventh child reported to have died. *The Guardian*.

Office for National Statistics. (2022). *Population estimates for England and Wales: mid-2022*.

PA Media. (2022, November 25). Surrey school pupil dies of bacterial infection with another in hospital. *The Guardian*. https://www.theguardian.com/uk-news/2022/nov/25/surrey-school-pupil-dies-of-bacterial-infection-with-another-in-hospital-report

Perrin, I. (2022, November 25). High Wycombe pupil dies after catching Strep A bacteria. *Bucks Free Press*.

RCGP. (2022a). *College issues advice on Strep A in joint statement with RCPCH and RCEM*. RCGP.Org.Uk. https://www.rcgp.org.uk/News/Joint-Strep-Statement#:~:text=We%27d like to reassure,Group A strep (iGAS).

RCGP. (2022b). *Worried parents need clear advice about when to seek medical help for their children with suspected Strep A, says College Chair*. RCGP.Org.Uk. https://www.rcgp.org.uk/News/Government-Messaging-Strep-Advice

Robertson, K. (2022, November 24). Six-year-old dies after rare bacteria outbreak at primary school. *The Metro*. Six-year-old dies after rare bacteria outbreak at primary school

Royal College of General Practice. (2024). *RCGP Public Health Data*. https://www.rcgp.org.uk/representing-you/research-at-rcgp/research-surveillance-centre/public-health-data

SkyNews. (2022, November 25). Six-year-old dies after Strep A bacteria outbreak at Surrey primary school. *Sky News*. https://news.sky.com/story/six-year-old-dies-after-strep-a-bacteria-outbreak-at-surrey-primary-school-12755138

Telegraph Reporters. (2022, November 25). Pupil, 6, dies from Strep A infection after outbreak at school in Surrey. *The Telegraph*. https://www.telegraph.co.uk/news/2022/11/25/strep-outbreak-ashford-surrey-child-death/

UKHSA. (2022a). *Group A Strep - What you need to know*. UKHSA.Blog.Gov.Uk. https://ukhsa.blog.gov.uk/2022/12/05/group-a-strep-what-you-need-to-know/

UKHSA. (2022b). *UKHSA update on scarlet fever and invasive group A strep*. https://www.gov.uk/government/news/ukhsa-update-on-scarlet-fever-and-invasive-group-a-strep-1#:~:text=Friday 3 March 2023,the peak in December 2022

1. ISO: International Organisation for Standardisation [↑](#footnote-ref-1)
2. NFR: No further deaths reported [↑](#footnote-ref-2)
