# Supplementary material for: Dynamics of public health messaging and healthcare activity in children during the 2022 iGAS surge: an observational study in England
Source: J Public Health (Oxf). 2026 Jan 12;48(1):281–90. doi: 10.1093/pubmed/fdaf163 (PMC13017340; doi:10.1093/pubmed/fdaf163)
Supplement: Supplementary_material_D_fdaf163 [file supplementary_material_d_fdaf163.docx]

# Supplementary material D: Specific advice given to parents on 2^nd^ December 2022 and repeated on 5^th^ December **^[[1]](#footnote-1)^**

**Contact NHS 111 or your GP if:**

- Your child is getting worse
- Your child is feeding or eating much less than normal
- Your child has had a dry nappy for 12 hours or more or shows other signs of dehydration
- Your baby is under 3 months and has a temperature of 38C, or is 3 to 6 months and has a temperature of 39C or higher
- Your baby feels hotter than usual when you touch their back or chest, or feels sweaty
- Your child is very tired or irritable

1. (UKHSA, 2022b) On 2 December (ISO week 48), UKHSA released 2-page public communication reporting five deaths in children under ten and advising parents or guardians to "contact NHS 111 or your GP if you suspect your child has scarlet fever." The symptoms of scarlet fever were described but no clinical images were included, and the press release gave this list of features for parents to use as triggers to contact health services. [↑](#footnote-ref-1)
