# Supplementary material for: Dynamics of public health messaging and healthcare activity in children during the 2022 iGAS surge: an observational study in England
Source: J Public Health (Oxf). 2026 Jan 12;48(1):281–90. doi: 10.1093/pubmed/fdaf163 (PMC13017340; doi:10.1093/pubmed/fdaf163)
Supplement: Supplementary_material_E_fdaf163 [file supplementary_material_e_fdaf163.docx]

# Supplementary material E: Area chart of weekly diagnosis rate of tonsillitis & acute pharyngitis and strep throat & peritonsillar disease in General Practice between November-January each year from 2017/8 to 2022/3

Alt Text: Graphical illustration of primary care attendances with tonsillitis and acute pharyngitis, or strep throat and peritonsillar infection between November-January each year from 2017/8 to 2022/3.
