# Supplementary material for: Dynamics of public health messaging and healthcare activity in children during the 2022 iGAS surge: an observational study in England
Source: J Public Health (Oxf). 2026 Jan 12;48(1):281–90. doi: 10.1093/pubmed/fdaf163 (PMC13017340; doi:10.1093/pubmed/fdaf163)
Supplement: Supplementary_material_F_fdaf163 [file supplementary_material_f_fdaf163.docx]

# Supplementary material F: A&E attendances by department in England for the months of winter 2022/23 compared to average attendances for the same months from 2018-2022 (excluding winter 2020-21) ^[[1]](#footnote-1)^

| Month | ED Department Type | | | |
| --- | --- | --- | --- | --- |
|  | Major | Single Specialty | Minor Injury/Other | Total |
| Mean monthly attendance  November-January *^[[2]](#footnote-2)^* | 1,307,307 | 44,188 | 682,368 | 2,033,862 |
| November 2022  (compared to mean) | 1,395,183  (+7%) | 42,644  (-3%) | 740,977  (+9%) | 2,178,804  (+7%) |
| December 2022  (compared to mean) | 1,437,681  (+10%) | 36,592  (-17%) | **819,806**  **(+20%)** | 2,294,079  (+18%) |
| January 2023  (compared to mean) | 1,243,898  (-5%) | 42,076  (-5%) | 682,364  (0%) | 1,968,338  (-3%) |

1. [https://www.england.nhs.uk/statistics/statistical-work-areas/ae-waiting-times-and-activity/](https://eur01.safelinks.protection.outlook.com/?url=https%3A%2F%2Fwww.england.nhs.uk%2Fstatistics%2Fstatistical-work-areas%2Fae-waiting-times-and-activity%2F&data=05%7C02%7Calexandra.creavin%40ukhsa.gov.uk%7C3bf7eddce58b42c9fe5d08dc6ab50414%7Cee4e14994a354b2ead475f3cf9de8666%7C0%7C0%7C638502572905196138%7CUnknown%7CTWFpbGZsb3d8eyJWIjoiMC4wLjAwMDAiLCJQIjoiV2luMzIiLCJBTiI6Ik1haWwiLCJXVCI6Mn0%3D%7C0%7C%7C%7C&sdata=SDIFooyS%2Bk9MDNEn0SljzEWqenS%2B1245GSS8%2BwAzmpg%3D&reserved=0) [↑](#footnote-ref-1)
2. 2018/19, 2019/20, 2021/22. Winter 2020/21 excluded. [↑](#footnote-ref-2)
